# Fever of unknown origin and Q-fever: a case series in a Bulgarian hospital

**DOI:** 10.22088/cjim.10.1.102

**Published:** 2019

**Authors:** Magdalena Baymakova, Georgi T. Popov, Radina Andonova, Valentina Kovaleva, Ivan Dikov, Kamen Plochev

**Affiliations:** 1 Department of Infectious Diseases, Military Medical Academy, Sofia, Bulgaria

**Keywords:** Fever of unknown origin (FUO), Coxiella burnetii, Q-fever, Bulgaria.

## Abstract

**Background:**

Fever of unknown origin (FUO) is a perplexing medical problem. The causes for FUO are more than 200 diseases. The aim of the study was to present human clinical cases of* Coxiella burnetii *infection debuting as FUO.

**Methods:**

The following methods were conducted in the study: literature search, laboratory, imaging, and statistical methods. Criteria of Durack and Street were applied for FUO definition. For the etiological diagnosis indirect immunoenzyme assay (ELISA) for antibodies detection against *Coxiella burnetii* was used (cut-off = 0.481–0.519).

**Results:**

From 2008 until 2015, nine patients with FUO caused by* C. burnetii *were hospitalized at the Military Medical Academy of Sofia. Male gender was predominant (male/female – 77.8% /22.2%), mean age was 48.78±14.52 years (range: 26–67), hospital stay was 9.78±2.95 days (range: 5–15), fever duration was 54.33±56.23 days (range: 21–180). Laboratory investigations estimated the elevation of erythrocyte sedimentation rate 49.11±31.74mm/h (95%CI = 13.09–111.31), C-reactive protein 37.68±37.62mg/L (95% CI = 36.07–111.42) and fibrinogen 5.69±1.59g/L (95% CI=2.57–8.81). The mean values of liver enzymes were in reference range. Among imaging tests, abdominal ultrasound and X-ray demonstrated 33.3% contribution to the final diagnosis. Transthoracic echocardiography found 22.2% contribution. Serological methods presented 100% contribution.

**Conclusion:**

*C. burnetii* infection was accepted as a final diagnosis among 9 patients with FUO based on the integrated information from the applied methods. Active search and establishment of this pathogen among FUO should lead to avoiding potential complications and consequences in case of untreated patients infected with* C. burnetii.*

For the first time, fever of unknown origin (FUO) was defined in 1961 by Robert G. Petersdorf and Paul B. Beeson (1). They gave the following definition of FUO: (a) fever higher than 38.3^0^С (101^0^F) in several measurements; (b) duration of fever for at least 3 weeks; (c) diagnosis remains unclear after a week of active diagnosis in a hospital (1). In 1991 two other American researchers David T. Durack and Alan C. Street changed the third criterion for FUO in the following form: „the diagnosis remains unspecified after 3 outpatient visits or 3 days in hospital” (2). Dutch scientist Chantal P. Bleeker-Rovers has presented a new definition of FUO in 2007 (3)*. Professor Bleeker-Rovers retained the first two criteria and removed the third criterion (*3*, *4*). The researcher added a new third criterion – exclusion of immunocompromised individuals and a fourth criterion for a mandatory diagnostic investigation (*3*, *4*)*. 

Reasons for FUO are arranged in five groups: (a) infectious diseases, (b) neoplasms, (c) non-infectious inflammatory diseases, (d) miscellaneous and (e) undiagnosed (5, 6). Until 2012 in Bulgaria, there was little scientific work and few articles on FUO (7-11). Since 2012, researchers from Department of Infectious Diseases, Military Medical Academy, Sofia (Bulgaria) have been starting comprehensive projects in FUO (12, 13). They did retrospective and prospective FUO studies for Bulgaria (14-21). For the period 2008 to 2015 in Bulgaria, the registered cases of human Q-fever were 158 (0.27 cases per 100 thousand population; 95% CI = 16.08–23.41) (22). The highest morbidity of human Q-fever was observed in Plovdiv district (23). The aim of the study is to present a case series of *Coxiella burnetii* infection presenting as FUO in a Bulgarian hospital. 

## Methods

**Study design and participants:** An observational study was conducted between January 2008 and March 2015 at the Department of Infectious Diseases, Military Medical Academy, Sofia (Bulgaria). The definition of Durack and Street for FUO was applied in the present study (2). Patients older than 18 years were enrolled (2). Measurements of body temperature were performed with a digital thermometer MC-343F-E (OMRON Flex Temp Smart; OMRON Healthcare Co., Ltd., Ukyo-ku, Kyoto, Japan), accuracy of measurement ±0.1^0^С (range: 32.0^0^С–42.0^0^С). The thermometry was realized in the axillary area under the supervision and control of a physician or nurse at an ambient temperature of 20.0^0^C to 28.0^0^C. 

**Laboratory and diagnostic tests:** Various laboratory tests have been applied during the diagnostic process: WBC, ESR, Fibrinogen, CRP, AST, ALT, GGT, AP and other laboratory indicators. Depending on the medical history and physical examination and diver imaging studies were carried out: abdominal ultrasound, x-ray, transthoracic echocardiography, computed tomography. Etiological diagnosis included culture methods, serology tests and molecular assays. *Coxiella burnetii* phase 1 IgA/IgG and *Coxiella burnetii* phase 2 IgG/IgM antibodies were detected in serum by indirect immunoenzyme assay (SERION ELISA classic, Virion/Serion, Würzburg, Germany), and according to the manufacturer’s instructions. *Coxiella burnetii* phase 1 IgA/IgG sensitivity 94.2%, specificity 96.2%; *C. burnetii* phase 2 IgG sensitivity 93.4%, specificity 98.5%; and *C. burnetii* phase 2 IgM sensitivity 94.4%, specificity >99%. The cutoff-evaluation of *C. burnetii* phase 1 IgA and IgG, respectively *C. burnetii* phase 2 IgM were calculated for each sample according to the manufacturer’s prescription and varying between from 0.481 to 0.519. *Coxiella burnetii* phase 1 IgA/IgG, resp. *C. burnetii* phase 2 IgM were defined as positive when optical density (OD) sample is more than 10% over OD cutoff, as negative when OD sample is more than 10% under OD cutoff, and as borderline when OD sample +/– 10% of OD cut-off. SERION ELISA classic *C. burnetii* phase 2 IgG was expressed in U/ml titer using a mathematical calculation and was defined as positive when the titer was >30 U/ml, as negative when the titer was <20 U/ml, and as borderline when the titer was 20–30 U/ml. 

**Statistics:** Statistical analysis was performed by Excel 2007 (Microsoft, Redmond, Washington, USA) and SPSS Statistics 19.0 (IBM Corp., Armonk, New York, USA). When p-value <0.05 the result was statistically significant. 

**Ethics:** The medical procedures of this study were approved by the Local Ethics Committee of Military Medical Academy, Sofia, Bulgaria (3 St. Georgi Sofiyski Str., 1606 Sofia). 

## Results

In the period of January 2008 to March 2015, one hundred and thirteen patients with FUO were investigated at the Department of Infectious Diseases, Military Medical Academy, Sofia (Bulgaria). The distribution of etiological groups was: 58.4% infections, 4.4% neoplasms, 13.3% non-infectious inflammatory diseases, 5.3% miscellaneous and 18.6% undiagnosed cases. 

After a comprehensive diagnostic process nine patients were classified as *C. burnetii *infection. They had serological data for Q-fever. The final diagnosis was determined by medical history, laboratory data and positive serological results. 

We analyzed the epidemiological, clinical and laboratory parameters in the group of cases with diagnosed Q-fever. Male gender was predominant (male/female – 77.8%/22.2%), mean age was 48.78±14.52 years (range: 26–67), hospital stay was 9.78±2.95 days (range: 5–15), fever duration was 54.33±56.23 days (range: 21–180). Clinical data of patients with *C. burnetii* infection were presented in table 1. Laboratory investigations estimated the elevation of ESR 49.11±31.74mm/h (95% CI=13.09–111.31), CRP 37.68±37.62mg/L (95% CI=36.07–111.42) and fibrinogen 5.69±1.59g/L (95% CI=2.57–8.81). Laboratory parameters were shown in table 2. The mean values of liver enzymes were in reference range. The serological results for *C. burnetii* infection were presented in table 3. The abdominal ultrasound and x-ray demonstrated 33.3% contribution to the final diagnosis. Transthoracic echocardiography found 22.2% contribution. Serological methods presented 100% contribution. 

**Table 1:** Clinical data of patients with *Coxiella burnetii* infection presenting as FUO

**Patient**	**Sex/Age**	**Sweats**	**Chills**	**Fatigue**	**Cough**	**Arthralgias**	**Animal contact**	**Fever, duration** (days)
1	M*/60	No	Yes	No	No	No	No	180
2	M/50	No	No	Yes	Yes	No	No	21
3	M/67	Yes	Yes	Yes	No	No	No	30
4	F**/26	No	Yes	Yes	Yes	Yes	Yes	21
5	F/53	No	No	Yes	No	No	No	119
6	M/33	No	Yes	Yes	No	Yes	No	30
7	M/57	No	No	Yes	No	Yes	No	30
8	M/60	Yes	Yes	No	Yes	Yes	No	30
9	M/33	Yes	Yes	No	Yes	No	Yes	30

* M: Male

** F: Female

**Table 2:** Laboratory data of nine patients with Q-fever presenting as FUO

**Patient**	**WBC** **(3.5-10.5 x10** ^9^ **/L)**	**ESR** **(≤ 20 mm/h)**	**Fibrinogen** **(2.0-4.5 g/L)**	**CRP** **(0.0-5.0 mg/L)**	**AST** **(5-40 IU/L)**	**ALT** **(5-40 IU/L)**	**GGT** **(10-50 IU/L)**	**AP** **(64-300 IU/L)**
1	7	39	7	ND	18	16	21	161
2	7	36	8	64	15	15	46	ND
3	3	95	5	17	110	58	135	418
4	7	5	3	2	39	13	22	121
5	8	87	6	42	14	27	28	270
6	13	82	ND	107	38	65	47	330
7	9.3	30	5.1	7.7	13	16	38	120
8	7	48	5.7	60	19	28	25	213
9	6	20	ND	1.7	41	96	25	ND

WBC: white blood cells; ESR: erythrocyte sedimentation rate; CRP: C-reactive protein; AST: aspartate transaminase; ALT: alanine transaminase; GGT: gamma-glutamyl transferase; AP: alkaline phosphatase; ND: no data available

**Table 3:** Serological results of *Coxiella burnetii* in Bulgarian patients with FUO

**Patient**	**Phase 1 IgA**		**Phase 1 IgG**		**Phase 2 IgG**		**Phase 2 IgM**	
	**Cut-off**	**Result**	**Cutoff**	**Result**	**Cutoff ** *	**Result (U/ml)**	**Cutoff**	**Result**
1	0.497	0.500	0.512	0.515	NA	67	0.486	0.632
2	0.502	0.509	0.491	0.498	NA	123	0.507	0.712
3	0.485	0.481	0.499	0.495	NA	97	0.489	0.698
4	0.493	0.485	0.514	0.506	NA	79	0.481	0.654
5	0.498	0.503	0.482	0.487	NA	169	0.512	0.747
6	0.511	0.519	0.493	0.501	NA	189	0.517	0.769
7	0.484	0.481	0.515	0.512	NA	85	0.486	0.683
8	0.519	0.512	0.491	0.484	NA	158	0.501	0.734
9	0.507	0.502	0.483	0.478	NA	173	0.519	0.758

* Positive: >30 U/ml; Negative: <20 U/ml; Borderline: 20–30 U/ml; NA: not applicable

## Discussion

Infections are the most common causes of FUO. The leading infectious diseases are tuberculosis, infective endocarditis and abscess (24, 25). Q-fever is a rare cause of FUO. Xiao-chun Shi et al. reported 0.1% cases of *C. burnetii* infection presenting as FUO in a study based on total 997 FUO patients (26). 

Researchers from Greece announced 2.9% cases of Q-fever in group of infectious diseases [n (ID)=34] among one hundred and twelve patients with FUO (27). Mete et al. found 1.0% cases of *C. burnetii* debuting as FUO in population of 100 cases (28). Investigators from United Kingdom presented 4.3% cases of Q-fever among twenty three patients with FUO (29). 

Ko et al announced seven cases of acute infection with *C. burnetii* in Taiwan (30). Ben-Baruch et al from Israel found 9.1% cases of Q-fever in the group of diagnosed infectious diseases among FUO population with 75 participants [n(ID) = 11; n(FUO) = 75] (31). French investigator Thierry Zenone presented 3.7% cases of *C. burnetii* infection in the group of diagnosed patients [n(Diag) = 107; n(Undiag) = 144] (32). In the present study, we reported nine cases (9/113; 7.96%) of Q-fever presenting as FUO. In comparison with other studies, the announced cases of *C. burnetii *infection are high. 

Divers reasons could influence this result. First, the geographic location of Bulgaria and local climate create a good condition for the development of this infection. Second, the hygiene requirements of livestock farms (cows, sheep, goats) are very often lowered. Third, veterinary control of dairy products (milk, cheese, yellow cheese, butter) is not always enough protective in the rural area of Bulgaria. Fourth, weather conditions for the development of ticks (as vectors for transmission of *C. burnetii*) are appropriate in our country. 

In the present study, the mechanism of infection is unclear, the epidemiological data are not enough to summarize the potential way of transmission. All this require further researches in the field of Q-fever and FUO in Bulgaria. In conclusion, the diagnostic detection of any case of FUO is a serious challenge for the physician. Q-fever as a cause of FUO is a reason, requiring a high attention in the diagnostic process. 

The scientific data for the connection between Q-fever and FUO are small. All these facts are a start point for further investigations. 
